# Ferns as facilitators of community recovery following biotic upheaval

**DOI:** 10.1093/biosci/biae022

**Published:** 2024-03-27

**Authors:** Lauren Azevedo-Schmidt, Ellen D Currano, Regan E Dunn, Elizabeth Gjieli, Jarmila Pittermann, Emily Sessa, Jacquelyn L Gill

**Affiliations:** Department of Entomology and Nematology, University of California Davis, Davis, California, and Climate Change Institute, University of Maine, Orono, Maine, United States; Department of Botany, Department of Geology and Geophysics, University of Wyoming, Laramie, Wyoming, United States; Natural History Museums of Los Angeles County, La Brea Tar Pits and Museum, Los Angeles, California, United States; New York Botanical Garden, Bronx, New York, United States; Department of Ecology and Evolutionary Biology, University of California Santa Cruz, Santa Cruz, California, United States; New York Botanical Garden, Bronx, New York, United States; Climate Change Institute, School of Biology and Ecology, University of Maine, Orono, Maine, United States

**Keywords:** ecosystem recovery, facilitation, paleontology, plant ecology, interdisciplinary science

## Abstract

The competitive success of ferns has been foundational to hypotheses about terrestrial recolonization following biotic upheaval, from wildfires to the Cretaceous–Paleogene asteroid impact (66 million years ago). Rapid fern recolonization in primary successional environments has been hypothesized to be driven by ferns’ high spore production and wind dispersal, with an emphasis on their competitive advantages as so-called disaster taxa. We propose that a competition-based view of ferns is outdated and in need of reexamination in light of growing research documenting the importance of positive interactions (i.e., facilitation) between ferns and other species. Here, we integrate fossil and modern perspectives on fern ecology to propose that ferns act as facilitators of community assemblage following biotic upheaval by stabilizing substrates, enhancing soil properties, and mediating competition. Our reframing of ferns as facilitators has broad implications for both community ecology and ecosystem recovery dynamics, because of ferns’ global distribution and habitat diversity.

Ferns are typically found in the understory, where they dominate humid, low-light, and often nutrient-poor environments (Sharpe et al. 2010). Nevertheless, ferns can also successfully colonize highly disturbed or primary successional landscapes (e.g., newly exposed rock or mineral soil). Prior reviews have noted the adaptive capacity of ferns in successfully colonizing new environments including their wide physiological tolerances (Anderson 2021), adaptability to abiotic stress (Krieg and Chambers 2022), and dispersal capabilities (Jones et al. 2006, Thomas and Cleal 2022). In some cases, this adaptive capacity is thought to provide a competitive advantage following biotic upheaval, giving ferns a reputation as “disaster taxa” for their ability to quickly colonize and thrive following severe disturbance events. However, this competition-based framework overlooks the role ferns have played in promoting ecosystem recovery (Bulleri et al. 2016, Oreja et al. 2020), challenging a “disaster taxon” model that fails to account for positive interactions such as facilitation. In neoecology (i.e., the ecology of modern timescales), the ability of species to ameliorate environmental stressors via facilitation (*sensu* Stachowicz 2001) has been recognized for decades but is often overlooked by studies of community assembly and dynamics (Bruno et al. 2003). For example, nurse logs provide nutrients, moisture, and substrate that many species of trees require for regeneration (Oreja et al. 2020, Decombeix et al. 2021) and are a classic example of the importance of facilitation in forest regeneration. Meanwhile, paleoecology (i.e., the ecology of the fossil record) has largely ignored the role of facilitation (Valiente-Banuet et al. 2006, Decombeix et al. 2021), despite the existence of significant biological upheaval events such as mass extinctions, which provide ideal study systems for investigating the importance of positive interactions during the recovery of biodiversity following global perturbations.

To address this knowledge gap, we integrate neo- and paleoecological perspectives to propose a new model of ferns as facilitators following biotic upheaval. We first summarize the growing body of recent literature suggesting that ferns are facilitative following disturbance and, therefore, play an important role in ecosystem recovery by increasing soil nutrient quality (Lyu et al. 2019), shading newly growing species and increasing soil moisture or water availability (Gould et al. 2013), improving microclimates, altering microbial and macrofaunal communities, or influencing plant community assembly (table 1; Yang et al. 2021). Given these contemporary observations, we hypothesize that ferns act as facilitators rather than merely as successful pioneer taxa, following disaster events. We propose that ferns ameliorate their local environments, facilitating post-disaster recovery by making conditions more favorable for the recovery of other species that, in turn, promote the very high-moisture and low-light environments where many fern species thrive. By integrating modern ecological theory with deep-time paleontological thinking, our novel framework reimagines ferns as facilitators of community assembly. This framework, which envisions ferns as more than superior competitors, may help us to understand how terrestrial ecosystems recovered following some of the greatest challenges in Earth's history.

**Table 1.** Summary of various mechanisms by which ferns benefit secondary succession within ecosystems.

**Mechanism**	**Benefit to secondary succession**	**Citation**
Reduce competition	Tree ferns reduce available space for other conifer and angiosperm trees to colonize, decreasing overall competition for canopy producing trees. Ferns (*Dicranopteris* species) can outcompete invasive or alien species, sometimes not allowing invasive or alien communities to establish.	Brock et al. 2019, Yang et al. 2021, Yuan et al. 2019
Increase soil organic carbon, nitrogen, and phosphorus	Increase overall soil nutrient content because of fern detrital input was greater post deforestation benefiting successional communities.	Lyu et al. 2019, Zhao et al. 2012
Increase soil stability	Decrease soil runoff for successional species or communities.	Osman et al. 2021, Sanchez-Castillo et al. 2019, Yang et al. 2021
Aluminum accumulation	Al can negatively influence root development and nutrient uptake within plant communities and fern's ability to accumulate AI ameliorates the soil environment	Schmitt et al. 2017
Increase soil moisture and nitrogen	Greater germination of tree seedlings under fern thickets due to higher soil moisture, nutrients, and lower soil surface temperature.	Walker 1994, Gallegos et al. 2015
Reduce soil contamination and toxicity	Reduction of soil contaminants and toxicity allows for species to cultivate landscapes that have been previously disturbed, such as mining locations.	Yang et al. 2021
Recruitment	Ferns support seedling growth by providing important ground cover in addition to acting as nurse logs within some environments. This can be seen within extinct and extant ecosystems.	Gallegos et al. 2015, Decombeix et al. 2021, Zotz et al. 2021

*Note:* The hypothesized new framework of ferns as facilitators builds on the modern ecological research cited here.

## Ferns as disaster taxa

As defined by Rodland and Bottjer (2001, 95), *disaster taxa* are “opportunistic taxa, characterized by long evolutionary histories, that invade vacant ecospace during the survival interval, but which are forced into marginal settings during later phases of the recovery.” This definition implies that disaster taxa are competitively superior in the highly stressed conditions following biotic crises but are outcompeted by other taxa once environmental stress abates. Ferns’ ability to recolonize disturbed habitats is well-documented in both modern and geologic records, with the two most commonly cited examples being the 1980 Mount St. Helens eruption and the Cretaceous–Paleogene (K–Pg) asteroid impact that triggered the K–Pg mass extinction event 66 million years ago (mega-annum, Ma; Vajda and McLoughlin 2007, Schulte et al. 2010).

**Figure 1.**
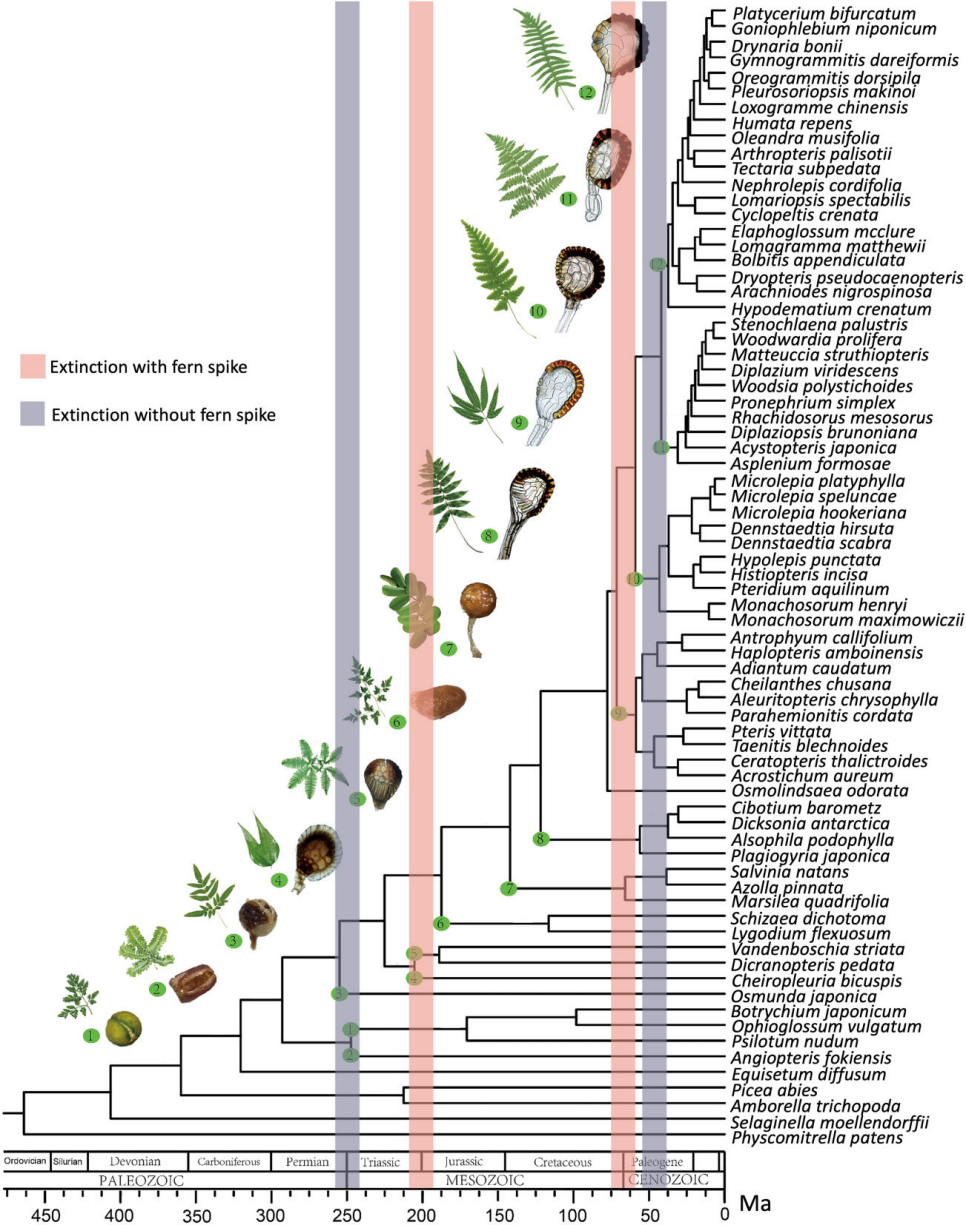
Time-calibrated fern phylogeny adapted from Shen and colleagues (2018). Major extinction events are shown as boxes centered around the Permian–Triassic (251 million years ago; in purple), Triassic–Jurassic (201.3 million years ago; in pink), Cretaceous–Paleogene (66 million years ago; in pink), and Eocene–Oligocene (33.9 million years ago; in purple). The dominance of ferns varies following these extinction events, with true fern spikes occurring during the Triassic–Jurassic and Cretaceous–Paleogene recoveries (the pink boxes), whereas the Permian–Triassic and Eocene–Oligocene events (the purple boxes) experienced a radiation but not dominance of ferns, and lack true fern spore spikes. Numbers in circles indicate corresponding lineages and representative leaves and sporangium with the same numbers.

The K–Pg asteroid strike (located in the modern-day Yucatán peninsula of Mexico) is recorded worldwide as an iridium-rich clay layer (Schulte et al. 2010). An anomalous fern spore spike just above the iridium layer was first identified within the Raton Basin, New Mexico (Orth et al. 1981), and was later found in additional sections (Tschudy et al. 1984). The fern spike was formally defined as a “palynological assemblage composed of 70 to 100% fern spores of a single species occurring within an interval 0–15 cm above the K–T boundary” (Nicholas and Johnson 2008, 18). Such spikes have also been identified within New Zealand (Vajda et al. 2001) and Japan (Saito et al. 1986), indicating global devastation of terrestrial vegetation and first recolonization of the post-K–Pg landscape by ferns (reviewed in Vajda and Bercovici 2014). In addition to the relatively rapid recovery of ferns represented by the fern spore spike itself, the K–Pg impact event may also have altered the trajectory of fern diversification. While ferns as a group are an ancient lineage (their earliest fossils date to the Devonian), most modern fern diversity at the species level has evolved in the 66 million years since the K–Pg impact (figure 1; Schuettpelz and Pryer 2009, Testo and Sundue 2016).

**Figure 2.**
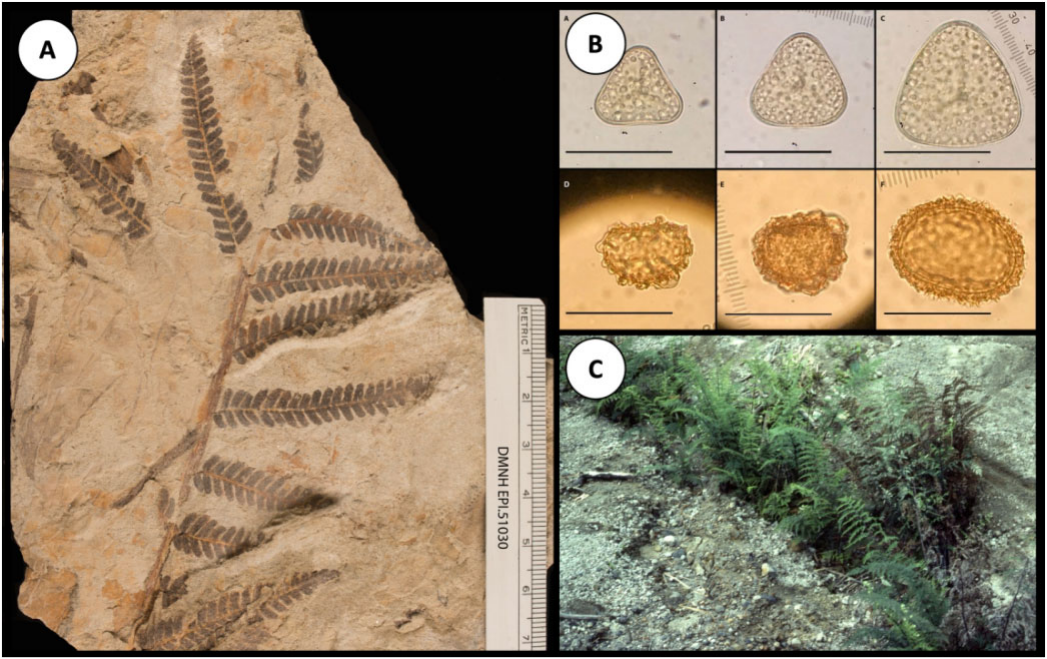
Examples of fern preservation within the fossil record as either compression macrofossils (a) from the Paleocene (***Cladophlebis*** sp.; DMNH EPI.51030) or microfossils (b) such as fern spores (Barrington et al. 2020). Within modern ecosystems ferns recolonize heavily disturbed landscapes such as the tephra of El Chichón in Southern Mexico (c). Photograph: RA Spicer, used with permission; Thomas and Cleal (2022).

Tschudy and colleagues (1984) introduced the idea of ferns as disaster taxa and proposed the Krakatau eruption and subsequent vegetation recovery as an analogue for the K–Pg boundary (for an example of ferns colonizing volcanos, see figure 2). They attributed ferns’ success in the earliest Paleocene to their wind-dispersed spores and tolerance of nutrient-poor soils, as well as the extinction of their competitors. In addition to their dispersal capabilities (Barrington 1993, Perrie and Brownsey 2007), fern spores also tend to form resilient, perennial spore banks in both temperate and tropical regions (Dyer and Lindsay 1992, Esaete et al. 2014, Berry 2019), and some species are able to tolerate soil conditions such as anoxia, heavy metal contamination, and increased salinity (Kachenko et al. 2007, Husby 2013), all of which would contribute to their post-disaster recovery and success. In modern systems, ferns have served as a primary carbon source for ecosystem regrowth after disturbance (Douterlungne et al. 2013). Finally, ferns are widely recognized for the frequency with which they undergo whole genome duplication to become polyploid (Wood et al. 2009), and researchers have increasingly posited a link between polyploidy, the capacity for ecological plasticity or adaptation, and diversification in plants (Cai et al. 2019, Sessa 2019), including in ferns (Berry 2022). These and other studies have demonstrated a tendency for polyploid lineages to be overrepresented following large-scale environmental change; given the frequency of polyploidy in ferns, it seems likely that their propensity for polyploidy may have played a role in their success after biotic upheavals, although additional tests of this hypothesis are needed.

Since the discovery of the K–Pg fern spike, paleobotanists have searched for similar patterns of recolonization and ecosystem recovery following global catastrophes and climate changes with varying success (e.g., Vajda and Bercovici 2014, Thomas and Cleal 2022). There is little evidence for fern dominance in recovery floras following the Permian–Triassic (P–T) extinction, the largest mass extinction in Earth's history, which has been attributed to a period of enhanced volcanism. Thomas and Cleal (2022) proposed that the absence of a P–T fern spike is because Permian ferns lacked the small sporangia and advanced dehiscence mechanisms present in more modern leptosporangiate ferns. A fern spike was documented in North America at the Triassic–Jurassic boundary (Olsen et al. 1990), when extensive volcanism in the Central Atlantic magmatic province triggered the end-Triassic extinction. Osmundaceous, marattalian, and schizaealean ferns were important, although not always dominant, components of end-Triassic extinction recovery floras in Greenland, Europe, Australia, and New Zealand (reviewed in Lindström 2016). These recovery floras were quickly replaced by gymnosperm-dominated floras typical of the Jurassic. A fern spike has also been observed in the Salisbury Embayment, in what is now Delaware, USA (Self-Trail et al. 2017) and in the North Sea (Eldrett et al. 2014), during the Paleocene–Eocene Thermal Maximum (PETM; Self-Trail et al. 2017), a geologically abrupt global warming event 56 million years ago that caused significant floral migration but little extinction (Wing and Currano 2013). However, similar patterns have not been noted elsewhere during the PETM (Korasidis et al. 2022).

## Fern functional traits and physiological resilience

**Figure 3.**
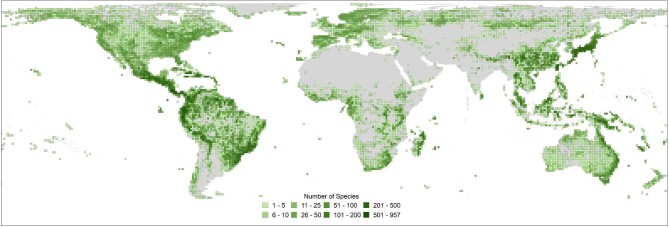
Map visualizing global fern species (class Polypodiopsida) richness using all known herbarium collection occurrences (after 1950; GBIF.Org 2023a 2), produced using geographic information system (GIS) mapping software ArcGIS Desktop Advanced 10.6 (ESRI 2018). Georeferenced data sets were spatially joined with a regular grid of 100,000 square kilometer cells (100 × 100 kilometers). The conjoined data set was extracted from the GIS and reconfigured to calculate and map the distinct species count per unique grid cell.

Contemporary fern communities are the legacy of species diversification (figure 1), especially following the dominance of closed canopies with the rise of angiosperms (Watkins and Cardelús 2012, Cai et al. 2021). This diversification has also allowed modern fern communities to colonize all major ecosystems (figure 3; Qian et al. 2022) because of a combination of unique physiological traits that are responsible for ferns’ success throughout the tropics, as well as in temperate and semi-arid environments (Sharpe et al. 2010); this suite of traits also supports their role as facilitators of community assembly.

Compared with seed plants, most fern sporophytes have a simplified body plan and slower metabolic rates that make them ideally suited to colonize dark and disturbed habitats such as those that resulted from the K–Pg impact (Walker and Sharpe 2010, Thomas and Cleal 2022). Relative to woody seed plants, fern sporophytes are metabolically inexpensive to build because they lack secondary growth (wood); the so-called trunks of tree ferns rely on a combination of rigid fibers, soft tissues, and turgor pressure for support (Pittermann et al. 2011, Mahley et al. 2018). Leaves emerge from a modified stem known as the rhizome, which serves as an anchor, a storage organ, and a meristem. Belowground, ferns’ roots are fine and shallow, and species may be adapted to myriad substrates, from rock to bark. Because respiration rates are higher in faster-growing plants (Lambers et al. 2008), a slow metabolism may be an advantage for colonizing low-light or resource-poor environments because such plants consume their carbon stores slowly and can effectively idle until conditions improve. Compared with conifers and woody angiosperms, which expend greater costs to produce seeds and wood, the generally simplified fern sporophyte body plan can support relatively consistent growth and spore production in resource-limited habitats.

The vast majority of ferns occupy low-light environments either within or under angiosperm canopies (Kawai et al. 2003, Schneider et al. 2004, Schuettpelz and Pryer 2009), and fern sporophytes would have been well-adapted for the cool, dark conditions that followed the K–Pg impact. A unique photoreceptor—neochrome—allows ferns to thrive in low light environments and may therefore have predisposed ferns to tolerate the presumably dark and cool K–Pg conditions (Li et al. 2014). Accordingly, fern photosynthetic rates rarely reach the levels of angiosperms in full sun and generally range from 2 to 10 micromols per square meter per second, whereas angiosperm leaves can reach over 50 umol/m^2^/s (Brodribb et al. 2007, Watkins et al. 2010, Pittermann et al. 2011). Although ferns are most often associated with mesic habitats, weedy ferns such as *Pteridium aquilinum* show remarkable plasticity when faced with habitat variation (Baer et al. 2020); a large number of fern species are surprisingly resilient to prolonged drought stress, with some species even thriving in arid, sunny environments (Hevly 1963, Riaño and Briones 2013). For example, many epiphytic ferns can experience seasonal and daily fluctuations in light and water availability and may have little to no organic substrate on which to anchor their sporophytes (Watkins and Cardelús 2012, Nitta et al. 2020). Such ferns may lack the water-holding capacity of soil that buffers terrestrial ferns during episodes of mild water scarcity, so their key strategy is to retain leaf water by reducing stomatal conductance and, therefore, minimizing water loss (Hietz and Briones 1998, Campany et al. 2021, Pittermann et al. 2023). However, some epiphytes, such as the well-studied *Pleopeltis* sp. (Polypodiaceae), are desiccation tolerant, meaning that they can lose over 95% of their metabolically active water and exist in a dormant stage until conditions improve (Oliver et al. 2000). Photosynthesis resumes quickly after rehydration, and recovery is facilitated by root pressure and capillarity, as well as the direct absorption of leaf water, a process known as *foliar uptake* (Holmlund et al. 2019, 2020, Prats and Brodersen 2021). Desiccation tolerance is an extreme and costly means to avoid mortality but is also indicative of ferns’ ability to thrive in harsh habitats.

In addition to their sporophytic traits, ferns (and lycophytes) are also unique among land plants in having gametophytes that are ecologically and nutritionally independent from the sporophyte parent. As with the other land plant lineages, the gametophyte is the site of sexual reproduction in ferns and is therefore a critical stage of the life cycle. Although they have a reputation for being delicate and ephemeral, fern gametophytes can be quite tolerant of stressful environments, including drought (Watkins et al. 2007, Pittermann et al. 2013, Chambers et al. 2017, López-Pozo et al. 2018). Several studies have demonstrated that fern gametophytes can have wider ecological tolerances than their sporophyte counterparts, and the gametophytes of a number of species have been shown to be capable of colonizing and persisting in microhabitats where sporophytes cannot (Watkins et al. 2007, Pittermann et al. 2013, Nitta et al. 2021; reviewed in Pinson et al. 2017). Fern gametophyte communities may also be structured differently than sporophyte communities (Nitta et al. 2017) and can demonstrate high functional diversity (Nitta et al. 2020). Taken together and considering the crucial importance of the gametophyte for sexual reproduction, it seems clear that the gametophyte generation must play an important role in how ferns respond to ecological disaster, although this has not yet been investigated directly.

**Figure 4.**
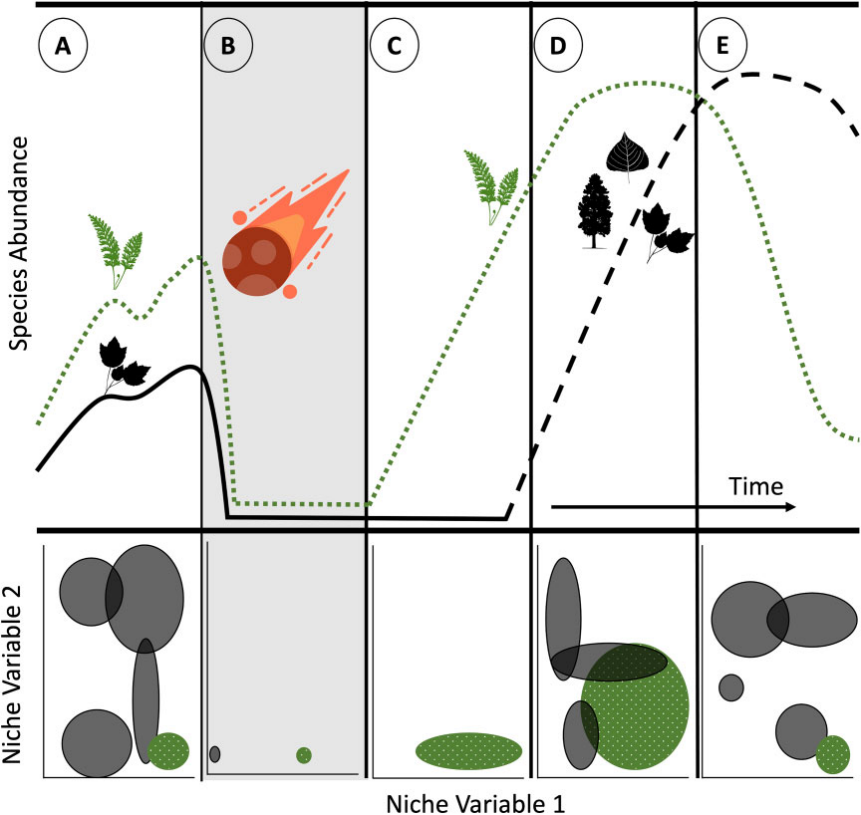
Conceptual figure showing fern facilitation before and after a biotic upheaval. Each column (a–e) shows how a theoretical community may respond at the level of species abundance (top) and the realized niche (bottom). In the top panels, time is on the ***x***-axis, and species abundance is shown on the ***y***-axis. Before the biotic upheaval, ferns (fern icons and green dotted line) are dominant on the landscape relative to seed plants (the black icons) with natural variability (a). Following the K–Pg (represented by the asteroid icon; b), fern and seed plant abundance dropped significantly, with widespread extinctions. However, because the habitat was ameliorated by fern facilitation, species began to reestablish (c–e; black dotted line). The presence of other species eventually decreased fern abundance, restricting them on the landscape (e). The black dotted line differs from the solid black line in panels (a) and (b) because of assumed differences in pre- and post-impact communities; in addition, it represents the positive influence ferns have on outside species that fosters both seed plant establishment and expands the available niche space available for ferns themselves. These changes in the environment are also shown in realized two-dimensional idealized niche space in the bottom panels with niche variable 1 on the ***x***-axis and niche variable 2 on the ***y***-axis. Non-fern plant communities are shown in various black circles and ferns are shown in green dotted circles with overlapping circles representing theorized overlap in niche space. Prior to the K–Pg ferns were limited in their realized niche space (a), minimally occurring on the landscape during the disturbance (b) but recolonized post-impact (c–e). Ferns were able to expand their niches because of a lack of competition (c) but eventually were restricted again on the landscape (d–e), highlighting the dynamic nature of community recovery.

The facilitative potential of ferns arises, in part, from their unique physiology, which enables them to colonize and transform devastated habitats under even the most climatically dismal conditions. A relatively inexpensive sporophyte body plan, coupled with a subterranean rhizome and slow metabolism, helps ferns to conserve their resources, whereas strategies to quickly ramp up photosynthesis after stress allow for quick recovery and growth when circumstances improve. The resilience of gametophytes and their ability to tolerate a wide range of ecological conditions would also promote recovery after large-scale environmental change. Slow nutrient release and light filtration may temper the initial success of seed plants in habitats dominated by ferns, but with the accumulation of organic matter and nutrients over time, ferns can be expected to thrive in a variety of conditions, even if they ultimately give way to the establishment of complex ecosystems that, together, form a mosaic of biodiversity (figure 4).

## Ferns as facilitators of community assembly and ecological recovery

The role of positive interactions among species has been increasingly acknowledged by ecological theory (albeit slowly; Bruno et al. 2003), and the field has shifted from a predominant focus on competition (e.g., Clements 1916) to a growing appreciation for the importance of processes such as facilitation and mutualism in driving species’ interactions and community dynamics (Koffel et al. 2021). Interactions germane to a facilitative framework have been documented across a range of communities, including bacterial assemblages (Piccardi et al. 2019), barnacle–snail systems (Cartwright and Williams 2012), hummingbird-pollinated plants (Bergamo et al. 2018), and carnivore communities (Périquet et al. 2015). A growing body of research indicates that ferns also play an important role as facilitators, due in part to their ability to alter and improve microhabitats following disturbance events (Wan et al. 2014).

Ferns improve environmental conditions in numerous ways (table 1), with many such changes occurring below-ground via soil modification. Ferns have been shown to alter microenvironments by ameliorating soil temperature, increasing moisture-holding capacity, reducing erosion, and increasing nutrient contents (Gallegos et al. 2015), which have been found to be important for enabling recolonization by secondary successional species (table 1). Some fern species also have the ability to stabilize soil through the pull-out force (i.e., the uprooting force) of their rhizomes (Sanchez-Castillo et al. 2019) and by decreasing runoff in denuded environments (Sanchez-Castillo et al. 2019, Osman et al. 2021, Yang et al. 2021). Fern communities therefore protect soil against erosion, allowing secondary successional species to recolonize. This is of particular importance when soil is newly developing after disturbance or disaster. Soil properties are also enhanced by fern communities, which increase organic carbon, nitrogen, and phosphorus through detrital inputs, specifically following disturbance such as deforestation (Zhao et al. 2012, Lyu et al. 2019). This is likely an artifact of ferns’ ability to colonize disturbed habitats first, leading to their detritus having the most impact on the recovering ecosystem.

The presence of ferns in a habitat can also increase soil moisture and decrease soil surface temperature (Walker 1994); further benefits to soil include the removal of contaminants, which greatly expedites recolonization by other plants. Some fern species have been found to remove heavy metals that accumulate in the soil such as aluminum (Schmitt et al. 2017) and arsenic (Chang et al. 2009), an advantageous trait because toxic levels of heavy metals have been recorded following the K–Pg impact (Erickson and Dickson 1987, Arenillas et al. 2018). The presence of metals such as aluminum negatively influences other plant species’ ability to uptake nutrients; therefore, because ferns reduce them from the soil, this could facilitate the establishment of more sensitive plants. Ferns also influence their habitats via seedling filtration, which mediates coexistence by minimizing competition among woody species, specifically between angiosperms and gymnosperms (Brock et al. 2018). Although the presence of some species of ferns have been found to suppress the regeneration of tree species (George and Bazzaz 1999a, 1999b, Levy-Tacher et al. 2015, Liu et al. 2022), in general, ferns support greater biodiversity in forest ecosystems by promoting establishment and minimizing competition (Brock et al. 2018, Yuan et al. 2019, Yang et al. 2021). By physically alternating their habitats, ferns influence community assembly via facilitation. Such mechanisms become especially important for ecosystem recovery in the aftermath of disturbance or disasters, because ferns promote the recruitment or growth of non-fern species (Valiente-Banuet et al. 2006, Bulleri et al. 2016). Such facilitative relationships have been documented within both terrestrial and aquatic environments and in a variety of organisms, from bryophyte and tree seedlings in temperate rainforests (Woods et al. 2021) to algae–barnacle interactions in coastal settings (Menge and Menge 2013).

Building from these contemporary examples, we hypothesize that the ferns represented by the K–Pg spore spike may also have been facilitators of ecosystem recovery in the aftermath of the K–Pg impact 66 million years ago. Under this model, ferns would have occupied a narrower realized niche space in the pre-impact landscape (figure 4a). The abiotic conditions following the K–Pg extinction event differed from the habitats in which most fern species are found today, and the ferns recorded by the fern spike were likely at the margins of their fundamental niches and physiological tolerances, although the realized niche may have been greater because of a release from competitive pressure. Following the biotic upheaval of the extinction event (figure 4b), ferns would have been some of the first plants to recolonize the barren landscape, possibly because of superior dispersal capabilities or because of physiological traits (discussed above) that allowed them to tolerate the extreme stress of the post-impact world, including low light conditions, acid rain, and denuded soils (figure 4c). Because ferns established in the absence of competition, they would have then modified these conditions via increasing soil moisture and nutrients and reducing erosion, therefore ameliorating the post-impact environment (figure 4d). Once established, fern sporophytes would have further improved soil stability and increased moisture and nutrient contents, expanding the availability of conditions for fern establishment and reproduction, especially for a greater diversity of species (fern gametophytes have a variety of environmental optima and niche breadths that vary among species; for a review, see Krieg and Chambers 2022). However, as a result of this facilitation, non-fern plant species (which were able to survive in isolated refugia or in the seed bank) would have then been able to expand, and ferns would eventually have become competitively excluded or marginalized within the very habitats they helped establish (figure 4e). Although fern abundances would have initially increased following the impact event (figure 4’s green line), non-fern species (the black dashed line) would have then out-competed ferns in high-light or disturbed environments, ultimately restricting most fern species back to the darker, more humid, nutrient-poor soils of the understory (figure 4e). Under this model, ferns would have played an essential role in ecosystem resilience and the recovery of biodiversity in the aftermath of one of the most significant biotic upheavals in Earth history.

## Recovery trajectories in modern versus fossil communities

If ferns are facilitators of community assembly following biotic upheaval, an improved understanding of their role in recovery dynamics—past and present—could help inform our understanding of the underpinnings of ecological resilience in the Anthropocene. Notably, there is an apparent orders-of-magnitude difference in the timescales of fern dominance in modern and fossil assemblages. Following modern upheavals such as the 1980 eruption of Mount St. Helens, ferns were the first to re-establish, but were followed by other plants within decades (Halpern et al. 1990, Titus et al. 2023). Recovery following the K–Pg impact was slower, because of the severity of the extinction event and the global impacts of the asteroid and its aftermath. According to some exceptionally well-dated records (Clyde et al. 2016), ferns likely dominated for ∼1000 years (but perhaps as long as 71,000 years because of dating uncertainty). Recovery was highly variable, both across clades and geographically, with some evidence that increasing distance from the impact site might have provided a buffer. For some groups, recovery was surprisingly quick; mammalian richness and body size recovery were estimated to have taken ∼100,000 years to reach pre-extinction levels (Lyson et al. 2019). In general, however, it took 10 million years for generic richness (i.e., the number of genera) to recover, which is comparable to or even faster than previous mass extinctions (Hull 2015). The temporal resolution of the fossil record influences our ability to determine true rates of recolonization, but by its very nature, a globally devastating event such as the K–Pg mass extinction had orders of magnitude greater influence on the timescale of ecosystem rebound than local events such as the 1980 Mount St. Helens eruption. Although the latter volcanic event influenced regional species pools in the short term (Dale et al. 2005), it is overshadowed in comparison to the global mass extinction and impact winter of the K–Pg aftermath, which decimated the global species pool and drove large-scale reductions in plant diversity (though few global extinctions at the family level; Wilf et al. 2023).

Compositional differences between fossil and modern floral communities could also contribute to differences in ecological recovery dynamics after biotic upheavals. Fern rhizomes (Spicer et al. 1985, Adams et al. 1987, Walker and Boneta 1995) and spores (Trejo et al. 2010) have been demonstrated to survive fires and other severe disturbance events, but this is also true for many members of non-fern clades, many of which evolved after the K–Pg (e.g., grasses). Although it's clear that ferns play a role in ecological recovery following modern-day disturbances such as fire, landslides, logging, or volcanic eruptions, community assembly and recovery dynamics in modern systems have almost certainly been shaped by the diversification of angiosperms throughout the Cenozoic. More work is needed to elucidate the role that ferns play in community assembly and resilience in both modern and fossil communities, which will allow us to better understand the degree to which fossil systems can be analogs for the present-day climate and biodiversity crises (i.e., conservation paleobiology; Dietl et al. 2015). For example, it would be helpful to know the degree to which recovery trajectories are influenced by the identity of pioneer taxa (i.e., priority effects), and whether, for example, ferns’ phylogenetic histories play a role in structuring recovery dynamics relative to angiosperms (Emerson and Gillespie 2008).

## Ecoevolutionary and conservation implications of ferns as facilitators

Although we have focused on a single clade (ferns), our framework has broadscale relevance for the understanding of facilitation in ecosystem resilience and recovery. As discussed above, ferns have played an important role in ecosystem recovery for millions of years and are able to tolerate a wide range of environments and stressors. Ferns are globally distributed (figure 3) and have an evolutionary history dating back to Devonian or earlier (Testo and Sundue 2016), allowing us to examine the importance of plant facilitation at broad spatiotemporal scales. If ferns are architects of recovery following upheaval, they may play an important role in ecological stability, which despite being widely studied remains poorly understood (Donohue et al. 2016, Domínguez-García et al. 2019). As we explore these relationships further, we may find even more positive interspecific interactions that have previously gone unrecognized but are nonetheless important for understanding biodiversity dynamics.

A growing body of research suggests that facilitation plays an important role in contemporary ecosystem and community processes, including stability and recovery (Horton and Van der Heijden 2008, Karst et al. 2015, Rodriguez-Ramos et al. 2021). As we have explored here, ferns may play a particularly important role in terrestrial ecosystem recovery following disturbance and upheaval across spatiotemporal scales. Furthermore, by viewing the fossil record through a facilitative lens, we can deepen our understanding of the drivers of long-term ecological and evolutionary dynamics in the aftermath of the K–Pg mass extinction, the most recent of Big Five mass extinctions widely recognized by paleontologists (Raup and Sepkoski 1982). As terrestrial ecosystems respond to a growing intersection of global changes, recognizing the role of facilitation in community assembly and ecological recovery becomes of critical importance for providing tools to support management efforts. By incorporating positive interactions into our ecological frameworks, we can better understand and support communities in an uncertain future.

## Author Biography

Lauren Azevedo-Schmidt (LAzSchmidt@ucdavis.edu) is affiliated with the Department of Entomology and Nematology at the University of California Davis, in Davis, California, and with the Climate Change Institute at the University of Maine, in Orono, Maine, in the United States. Ellen D. Currano is affiliated with the Department of Botany and the Department of Geology and Geophysics at the University of Wyoming, in Laramie, Wyoming, in the United States. Regan E. Dunn is affiliated with the Natural History Museums of Los Angeles County and of the La Brea Tar Pits and Museum, in Los Angeles, California, in the United States. Elizabeth Gjieli is affiliated with the New York Botanical Garden, in the Bronx, New York, in the United States. Jarmila Pittermann is affiliated with the Department of Ecology and Evolutionary Biology at the University of California Santa Cruz, in Santa Cruz, California, in the United States. Emily B. Sessa is affiliated with the New York Botanical Garden, in the Bronx, New York, in the United States. Jacquelyn L. Gill is affiliated with the Climate Change Institute, and with the School of Biology and Ecology at the University of Maine, in Orono, Maine, in the United States.
